# Current News

**Published:** 1896-06

**Authors:** 


### Current News.

### PENNSYLVANIA STATE DENTAL SOCIETY.

The next Annual meeting of the Pennsylvania State Dental Society will be held at Bellefonte, July 7, 1896, continuing for three days.

Victor S. Jones,
*Corresponding Secretary.*

Bethlehem, Pa.

### PENNSYLVANIA STATE DENTAL EXAMINING BOARD.

The Pennsylvania State Dental Examining Board will hold its next annual meeting at Bellefonte, Pa., July 7, 1896.

Wm. E. Magill, Erie, Pa.,
*President.*
J. C. Green, West Chester, Pa.,
*Secretary.*

### CHICAGO DENTAL SOCIETY.

List of officers of the Chicago Dental Society for 1896–97, elected at the annual meeting, held in Columbus Memorial Building, Tuesday evening, April 7, 1896:

President, Louis Ottofy; First Vice-President, J. E. Hinkins; Second Vice-President, H. A. Costner; Recording Secretary, A. H. Peck; Corresponding Secretary, Geo. B. Perry; Treasurer, E. D.

Swain; Librarian, H. A. Gunther; Member Board of Directors, G. H. Cushing; Board of Censors, G. T. Carpenter, B. D. Wikoff, G. W. Schwartz.

GEO. B. PERRY,
*Corresponding Secretary.*

---

### NORTH CAROLINA STATE DENTAL SOCIETY.

THE Twenty-second Annual Meeting of the North Carolina State Dental Society will meet at Morehead City, on June 17, 18, and 19, 1896. The first session of the Society will not be called to order until Wednesday morning, June 17.

The State Board of Dental Examiners will be held on Tuesday, the 16th, for the examination of all applicants for license. All persons desiring to come before the Board are requested to be present on Tuesday at ten o'clock, in order that all examinations may be completed by Wednesday morning.

J. E. WYCHE,
*Secretary.*

---

### AMERICAN DENTAL SOCIETY OF EUROPE.

THE American Dental Society of Europe will hold its twenty-first meeting at Dresden, Germany, August 3, 4, and 5, 1896. All members of the profession who plan to be in Europe at that time are cordially invited to attend.

Further information can be obtained of the president, Dr. John H. Spaulding, Paris, or of William A. Spring, 26 Christian Street, Dresden.

---

### THE FIFTY-FIRST ANNIVERSARY OF HORACE WELLS'S DISCOVERY.

THE fifty-first anniversary of the discovery of anæsthesia by Horace Wells was observed by the Quonehtacut Dental Club with a banquet at the Hotel Heublein, Hartford, on the evening of December 11, 1895. There were present Drs. E. S. Gaylord and D. A. Jones, of New Haven; C. Fones, A. C. Fones, and C. W. Strang, of Bridgeport; Chas. P. Graham, of Middletown; W. J. and W. H.

Rider, of Danbury; Edw. Prentis, of New London; J. Tenney Barker, of Wallingford; and Jas. McManus, Geo. L. Parmell, Henry and Charlas McManus, of Hartford.

The invited guests were Hon. O. Vincent Coffin, governor of Connecticut, and Mr. Charles T. Wells, the son of the discoverer of anæsthesia.

---

### INTERSTATE DENTAL MEETING.

A MEETING of the General Executive Committee of the Interstate Dental Meeting was held in Kansas City, at which each of the four States (Iowa, Nebraska, Kansas, and Missouri) was represented, and the following action taken: The place and time of the meeting was fixed at Excelsior Springs, Missouri, June 23–26, 1896. Dr. Henry J. McKellops was chosen supervisor of clinics, to be assisted by Dr. L. K. Fullerton, Iowa, Dr. O. M. Heustis, Nebraska, Dr. C. B. Reed, Kansas, and Dr. H. S. Lowery, Missouri.

Much enthusiasm was reported from all the States, and it is believed that this will be one of the greatest dental meetings ever held in the West.

J. P. ROOT,
*Chairman.*
S. C. A. RUBEY,
*Secretary.*

